# Electrospun Nanofiber/Textile Supported Composite Membranes with Improved Mechanical Performance for Biomedical Applications

**DOI:** 10.3390/membranes12111158

**Published:** 2022-11-17

**Authors:** Mohammed Jalalah, Adnan Ahmad, Asad Saleem, Muhammad Bilal Qadir, Zubair Khaliq, Muhammad Qamar Khan, Ahsan Nazir, M. Faisal, Mabkhoot Alsaiari, Muhammad Irfan, S. A. Alsareii, Farid A. Harraz

**Affiliations:** 1 Promising Centre for Sensors and Electronic Devices (PCSED), Advanced Materials and Nano-Research Centre, Najran University, Najran 11001, Saudi Arabia; 2 Department of Electrical Engineering, College of Engineering, Najran University, Najran 61441, Saudi Arabia; 3 Department of Textile Engineering, National Textile University, Faisalabad 37610, Pakistan; 4 Department of Materials, National Textile University, Faisalabad 37610, Pakistan; 5 Department of Textile & Clothing, Karachi Campus, National Textile University, Karachi 74900, Pakistan; 6 Department of Chemistry, Faculty of Science and Arts, Najran University, Najran 11001, Saudi Arabia; 7 Department of Chemistry, Faculty of Science and Arts at Sharurah, Najran University, Najran 11001, Saudi Arabia; 8 Department of Surgery, College of Medicine, Najran University, Najran 11001, Saudi Arabia

**Keywords:** PA nanofiber membrane, composite, nonwoven, woven, knitted, needleless electrospinning

## Abstract

Textile-supported nanocomposite as a scaffold has been extensively used in the medical field, mainly to give support to weak or harmed tissues. However, there are some challenges in fabricating the nanofiber/textile composite, i.e., suitable porous structure with defined pore size, less skin contact area, biocompatibility, and availability of degradable materials. Herein, polyamide-6 (PA) nanofibers were synthesized using needleless electrospinning with the toothed wheel as a spinneret. The electrospinning process was optimized using different process and solution parameters. In the next phase, optimized PA nanofiber membranes of optimum fiber diameter with uniform distribution and thickness were used in making nanofiber membrane–textile composite. Different textile fabrics (woven, non-woven, knitted) were developed. The optimized nanofiber membranes were combined with non-woven, woven, and knitted fabrics to make fabric-supported nanocomposite. The nanofiber/fabric composites were compared with available market woven and knitted meshes for mechanical properties, morphology, structure, and chemical interaction analysis. It was found that the tear strength of the nanofiber/woven composite was three times higher than market woven mesh, and the nanofiber/knitted composite was 2.5 times higher than market knitted mesh. The developed composite structures with woven and knitted fabric exhibited improved bursting strength (613.1 and 751.1 Kpa), tensile strength (195.76 and 227.85 N), and puncture resistance (68.76 and 57.47 N), respectively, than market available meshes. All these properties showed that PA nanofibers/textile structures could be utilized as a composite with multifunctional properties.

## 1. Introduction

Textile medical products are highly significant for the healthcare and hygiene sectors. Healthcare-associated infections (HAIs) are common adverse health events affecting hospital patients [1,2]. Therefore, a large number of healthcare and hygiene products are used in operating theaters and hospital wards [3]. Medical textile products used in hospital wards include bedding; clothing, mattress covers; incontinence products; clothes; wipes [1,4]; extracorporeal devices [5]; non-implantable materials such as plaster and pressure apparel [6]; and implantable materials such as sutures, synthetic ligament [7], the synthetic dermis [8], synthetic Lumina [9], eye-to-eye contact lenses, orthopedic implants synthetic joint capsules, synthetic bones [10], heart implants, vascular grafts, cardiovascular valves [11], and hernia meshes [12]. 

Surgical meshes have been used to resolve hernia problems since 1891. The research in this field has expanded due to multiple difficulties after surgeries, such as infections, fibrosis, adhesions, mesh rejection, and hernia recurrence. Scientists, scholars, and researchers developed various methods for a wide range of mesh materials, their analysis, and implementation with different porosity and fiber size. Several implantation and surgical methods have been established [13], such as biodegradable knitted meshes [14], tubular knitted meshes [15], PTFE-based knitted meshes [16], fibrous-based multilayer meshes [17], woven-based meshes [18,19], non-woven based meshes [20], and pad-like meshes [21]. However, achieving suitable porosity and material mechanical properties such as tensile tear strength, elongation, and stiffness is still challenging.

Electrospun-based nanofibers [22] offer a nano-level control of fiber mesh porosity and higher specific strengths. Shaohua et al. discussed a detailed review of how nanoscale fibers have gained much attention due to their superior performance compared to conventional electrospun fibers. These nanofibrous materials showed superior mechanical, electrical, biological, and optical properties in relation to microfibers and have a wide range of applications in biotextiles, tissue repair-regeneration, wearable textiles, and bioelectronics [23]. Liang et al. introduced the usage of a novel spinneret (annular), which resulted in high throughput nanofibers by needleless electrospinning. The novel electrospinning technique showed less solution evaporation because of the solution stored in the annular slit. Moreover, various jets formation was achieved at the edge of the spinneret. At 10% solution concentration, applied voltage at 60 kV, a flow rate of 14 mL/h, and a collection distance of 20 cm, the highest productivity (4.5 g/h) of nanofibers was achieved [24].

Some researchers worked on electrospun-based meshes; for example, Ebersole et al. developed an electrospun absorbable poly-caprolactone (PCL) scaffold in which two tale arrangements possessed elastic qualities and suture maintenance which were suitable for hernia fix applications [25]. K. Molnar et al. contemplated the 3D absorbable polyvinylalcohol (PVA) scaffold using reactive electrospinning for tissue-designed hernia substitution [26]. Pengbi Liu et al. studied warp-knitted-based hernia meshes with patterned nanofibers mats and found that mesh properties can be improved by suitable textile structure design [27]. Jason Chakroff et al. developed the bio-resorbable and non-resorbable polymers electrospun-based hernia mesh. Interestingly, it was found that the bio-resorbable polymers beat the no resorbable polymers in the mechanical tests [28]. B. Veleirinho et al. studied the PVA-based electrospun scaffold and showed that PVA hernia meshes did not support cell attachment in vitro [29]. M. Plencner et al. investigated the PVA molecular weight effect on needleless electrospinning [30]. Usman Ali et al. reported a comparative study on the needleless electrospinning of PVA nanofiber by using different spinnerets [31]. X Wang investigated the needleless electrospinning technique for the nanofibers’ scale production.

The authors fabricated the PVA nanofibers using multiple rings as the spinnerets and evaluated the effect of spinnerets on productivity and nanofiber morphology. It was stated the electric field mainly concentrated at the top side of all rings and directly affected productivity [32,33]. Fiber morphology and production rate both depended on the process variables such as the voltage, distance, and solution parameters, that is, concentration and molecular weight of the polymers [34,35,36]. The productivity of ring needleless electrospinning was almost 22 times higher than the conventional process of using two rings as a spinneret [37]. The main objective of this research study is the development of an electrospun-based nanofiber composite supported by different types of fabric (non-woven, woven, and knitted) to enhance biocompatibility, mechanical properties, and appropriate pore size. The electrospun membranes and resultant composites were characterized by SEM, stiffens, tensile, tear, and bursting strength properties. These properties were compared with two market available hernia mesh products (woven and knitted).

## 2. Materials and Methods

Polyamide-6 (MW: 150,000, density: 1.184 g/cm^3^) was purchased from Merck (made in Germany). Acetic acid was also purchased from Merck (made in Germany, Darmstadt, Germany); formic acid was purchased from Sigma Aldrich (St. Louis, MO, USA).

### Development of Nanofiber/Textile Composites

The development of electrospun nanofiber/textile composite was divided into 3 phases. The first phase objective was to obtain bead-free and smooth nanofibers from electrospinning. For this purpose, initially, pre-trials were carried out, varying the solution and process parameters such as solution concentration, applied voltage, and spinning distance to find out the optimized parameters. After pre-trials, these three factors were selected, i.e., polymer concentration (18, 20, and 22 *w*/*w*%), applied voltage (40, 45, and 50 KV) and spinning distance (16, 20, and 24 cm) because these factors have a prominent effect on results. A complete design of the experiment is given in Table 1. A homogeneous solution of PA with specific conc. of 18, 20, and 22% was prepared by dissolving PA polymer in acetic acid (50% *w*/*w*) and formic acid (50% *w*/*w*) solvents (*w*/*w*) solvents and continuously stirring at 1200 rpm for 24 h at 50 °C. The fabrication of PA nanofibers was carried out using a toothed wheel needleless electrospinning technique. The needleless electrospinning contains a toothed wheel, a grounded collector (rotating cylinder), a solution container that is made of Teflon, and a power supply (Gamma-high voltage DC power supply). The dimensions of the toothed wheel spinneret are as given thickness = 5 mm, exterior diameter = 60 mm, number of teeth = 14, and pitch = 13.45 mm. The other process parameters, such as toothed wheel rotation and collecting wheel rotation, were kept constant after the optimization of the process. The toothed wheel spinneret rotation was kept constant at 10 rpm, and the collecting cylinder speed was set at 50 rpm to collect uniform and even the nanofiber membrane. The applied voltage is connected to the toothed wheel spinneret, whereas the ground is connected to the rotating collector. The environmental conditions were also optimized and adjusted after pre-trials. The temperature and relative humidity were varied at 16 to 25 °C and 35 to 65% at the initial pre-trials during the electrospinning of PA6 nanofiber. After optimizing the environmental conditions, the temperature was set to 16 °C and 35% relative humidity and kept constant through the electrospinning of the PA6 nanofiber membrane. The membranes of different thicknesses were prepared initially, and the most suitable membranes with good tensile and bursting properties were selected for the development of composite structures.

In phase II, different fabrics, such as non-woven, woven, and knitted, were developed to support the nanofiber membranes. This process was completed in two steps. For fabric preparation, the spinning of polypropylene fibers (PP) was performed to develop PP yarn. Then, weaving was performed to prepare PP fabric. Next, knitting of the same PP yarn was performed to develop knitted fabric. Non-woven fabric was developed using PP staple fibers through needle punched non-woven fabric system.

After developing fabrics in phase III, the optimized nanofibers membranes with good tensile and bursting strength were attached to different textile structures through the calendaring process to fabric nanofiber nonwoven composite (NWC), woven composite (WC), and knitted composite (KC), respectively. The fabric substrate was sprayed with PA6 binder to obtain adhesion of the nanofiber membrane to the textile substrate. The calendaring speed was adjusted at 1.40 m/min, calendaring pressure at 12 bar, and the lower cylinder temperature was set at 120 °C. The combined layer of textile substrate and nanofiber membrane was passed between the calendars. The fabric substrate was toward a lower calendar (downward direction) during this process. The obtained composite of nanofiber membrane and fabric substrate has good adhesion properties and cannot peel off easily.

**Figure 1:**
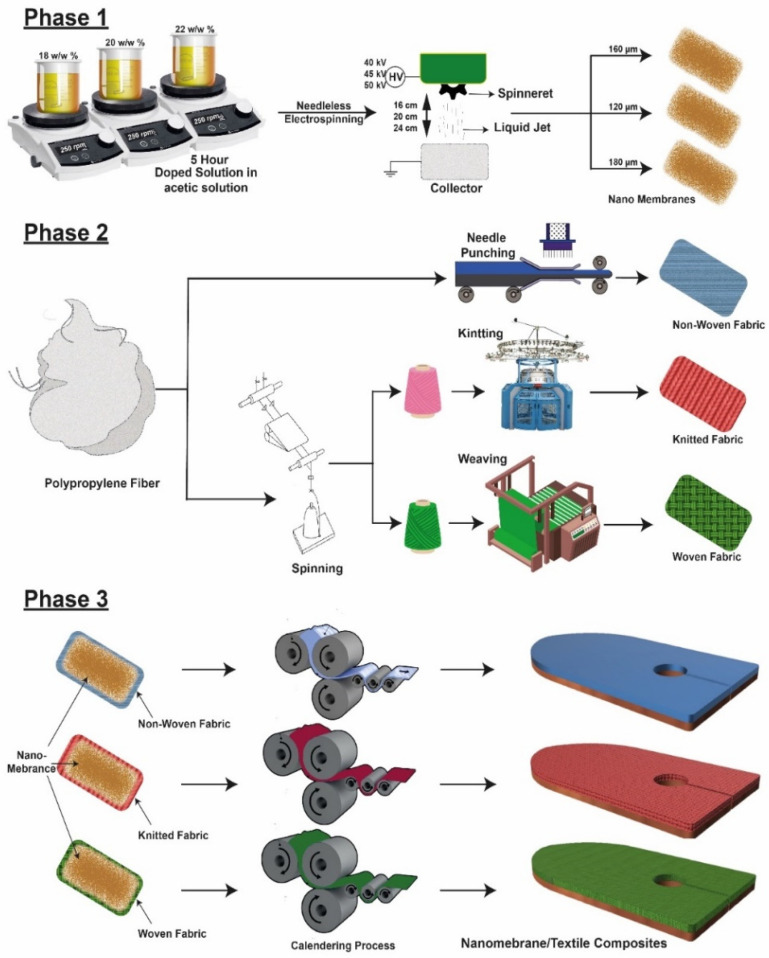
Illustration scheme of fabrication of nanofiber/textile composite.

For comparison purposes, two different hernia meshes were bought from the market. One was woven hernia mesh (MWM), and the second was knitted hernia mesh (MKM). Figure 1 shows the illustration of the fabrication of a nanofiber/textile composite. All the characterization of prepared nanofiber/textile composite and market-bought meshes were compared in this study.

## 3. Characterization

### 3.1. SEM Characterization of Electrospun Nanofibers

Scanning electron microscopy (Quanta 250 FEI) was used to observe morphologies of electrospun PA Nanofibers. The diameter of electrospun PA nanofibers was calculated from SEM images with the help of an image analyzer, “Image J”, and a minimum of 50 fibers were tested from each image.

### 3.2. Surface Area and Porosity

Brunauer–Emmett–Teller (BET) analysis was performed to measure the surface area and porosity of the PA6 nanofiber membrane through Brunauer–Emmett–Teller (BET) analysis (BET, Quantachrome Nova 2200e).

### 3.3. Mechanical Testing of Electrospun Nanofibers

The tensile strength of PA nanofibers was obtained with a (Tensiometric 2.5) single fiber strength tester with speed (10 mm/min) and gauge length (10 mm). Samples were tested at a temperature of 25 °C with 55% of relative humidity. Each sample was cut to dimensions 5 mm in width and 10 mm in length. Ten readings of each PA sample were tested.

The universal tensile strength tester was used to evaluate the strength of textile materials. The testing standard for fabric tensile strength using the strip method is EN ISO 13934-1:1999. The fabric strip was cut according to the dimensions 10″ × 3″ in both directions of testing fabric minimum of two sample warp directions and another in the weft direction. The tear strength tester was used to measure the tear strength. The working principle of this machine is a ballistic pendulum (Elmendorf) and a cutter that places a cut of 2 mm on the specimen. The testing standard for tear strength is EN ISO 13937-1:2000.

A bursting strength tester is an equipment that is used for measuring fabric bursting strength by applying pneumatic pressure on the sample until it bursts. Then, the reading is noted in Kpa. The testing standard for bursting strength is ISO 13938-2:1999. The testing standard for stiffness testing is ASTM-D4032.

## 4. Results and Discussions

### 4.1. Nanofibers Morphology

Fiber morphology is an important feature of the nanofiber, influencing the final nanofiber membranes’ performance. The solution concentration applied voltage and distance from nozzle tip to collector are primary factors for the structure and morphology of nanofibers fabricated from the multiple tooth wheel spinnerets. Initially, the electrospinning process was optimized with pre-trials under different process environmental conditioning. The temperature varied from 15 to 25 °C, and the relative humidity was 35 to 65%. At the high temperature (25 °C), the electrospinning process is not smooth due to the evaporation of the volatile solvent (acetic acid and formic acid) and fiber solidified before reaching the collector. Moreover, the solution concentration was also changed due to the evaporation of the solvent in the container during electrospinning. On the other side, the electrospinning of PA6 nanofiber was uniform and smooth at 16 °C temperature. Similarly, in the case of high relative humidity (65%), high moisture content between the spinneret and collector interacted with the polymer jets during its flight. This interaction caused the phase separation and solidified the PA6 nanofiber before reaching the collector, resulting in a coarser and uneven nanofiber membrane. Based on these trials, the chamber temperature was set at 16 °C, and relative humidity was kept at 35%. Solution concentration was also varied from 10 to 25%. It was observed that at low concentrations of 10 to 16%, beaded nanofibers were formed, while solution concentrations above 22% developed coarser ribbon-like nanofiber. Therefore, a solution concentration between 18 and 22% was selected for the further optimization of the PA6 nanofiber morphology. In the applied voltage, a minimum voltage of 30 kV is required to initiate the electrospinning process by using toothed wheel electrospinning. Uniform electrospinning of the PA6 nanofiber was observed for the voltage range from 35 to 45 kV. The spinning distance was also set between 16 and 24 cm after the pre-trials by analyzing the smooth electrospinning and evaporation of the solution.

#### 4.1.1. Impact of Polymer Concentration

**Figure 2:**
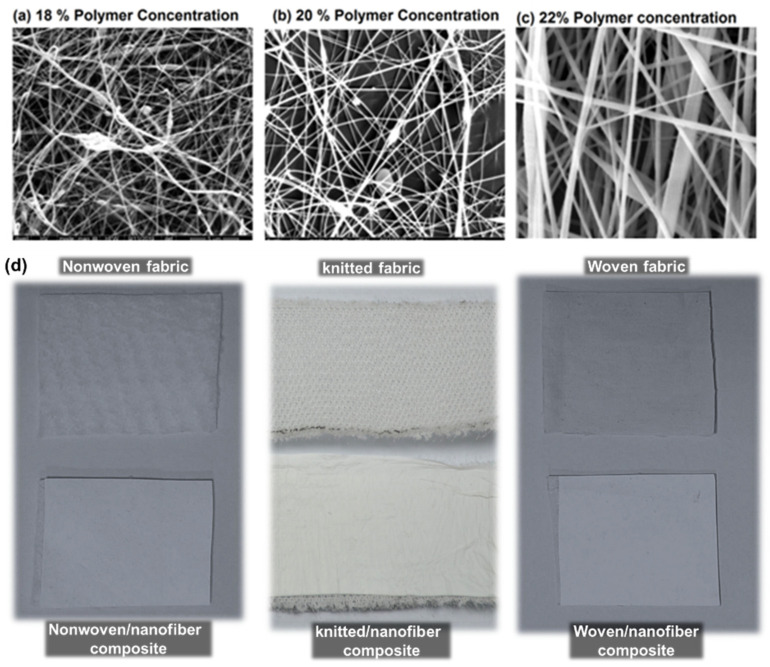
(**a**–**c**) SEM images of nanofibers diameter at different concentration levels 18, 20, and 22 *w*/*w*% with constant applied voltage 45 kV and constant collecting distance 20 cm; (**d**) actual photographic view of nonwoven, knitted, and woven composite.

**Figure 3:**
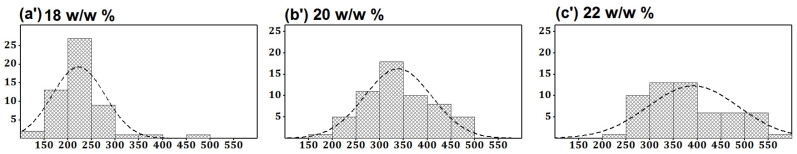
Histograms of nanofiber diameter at different concentration levels 18, 20, and 22 *w*/*w*% with constant applied voltage 45 kV and constant collecting distance 20 cm.

Figure 2 shows that finer nanofibers are formed at 18 *w*/*w*% polymer concentration. When the polymer concentration was increased to 20 *w*/*w*%, then the diameter of nanofibers was also increased. The further increase in concentration to 22 *w*/*w*% also enhanced the diameter of the nanofibers. The formation of beads at low concentrations was due to the polymer solution’s low viscosity; hence, surface tension dominated and led to the formation of beads instead of nanofibers. While at a higher concentration level, the more viscous polymer solution formed the continuous uniform nanofibers, as shown in Figure 3. The distribution of nanofiber diameter is presented in Figure 3 through the histogram. The distribution of the nanofiber diameter is narrow at 18 *w*/*w*% in comparison to the distribution of the diameter of the nanofibers. The average diameter of nanofibers found is 231 ± 65 nm, 343 ± 58 nm, and 418 ± 47 nm at a concentration level of 18, 20, and 22%, respectively. Figure 2d presents photographic images of the developed nonwoven, knitted, and woven composites with nanofiber membranes.

#### 4.1.2. Impact of the Applied Voltage

**Figure 4:**
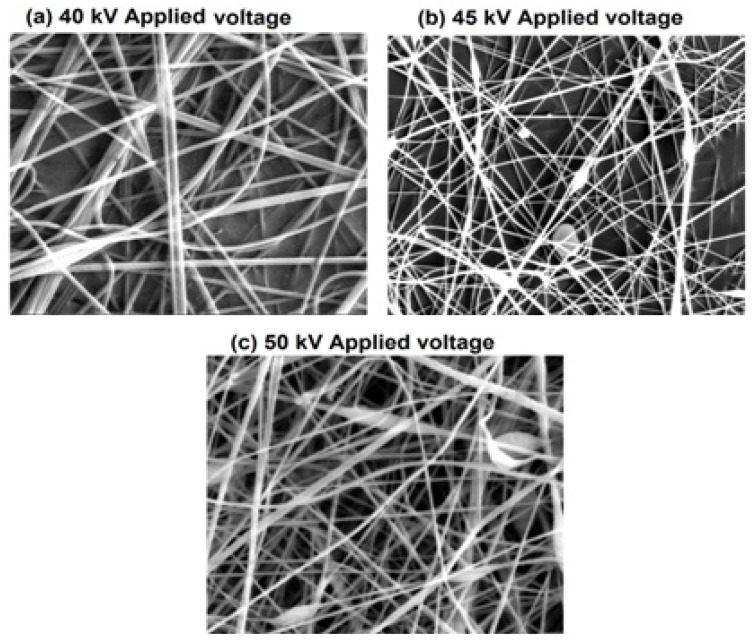
Histograms of nanofiber diameter at different concentration levels 18, 20, and 22 *w*/*w*% with constant applied voltage 45 kV and constant collecting distance 20 cm.

**Figure 5:**
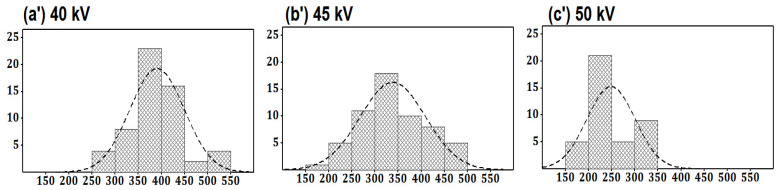
Histogram of diameter distribution at a different voltage with a constant concentration of 20% at a spinning distance of 20 cm.

Figure 4 and Figure 5 show the SEM images of nanofibers and the histogram of diameter distribution at a different voltage with a constant concentration of 20% at a spinning distance of 20 cm. The SEM image of PA nanofibers shows that the nanofibers’ diameter had a decreasing trend as the potential difference was increased from 40 kV to 50 kV while keeping other parameters constant. The average diameter of nanofibers was 383, 343, and 278 nm, formed at 40, 45, and 50 KV, respectively. It could be seen that the impact of applied potential difference was more prominent at the low concentration. The reason for shifting the trend of the diameter of nanofibers on the lower side of applying a higher voltage is due to the generation of stronger electrostatic force at charged jets, hence resulting in a narrowing diameter of the nanofibers.

#### 4.1.3. Impact of Collecting Distance

**Figure 6:**
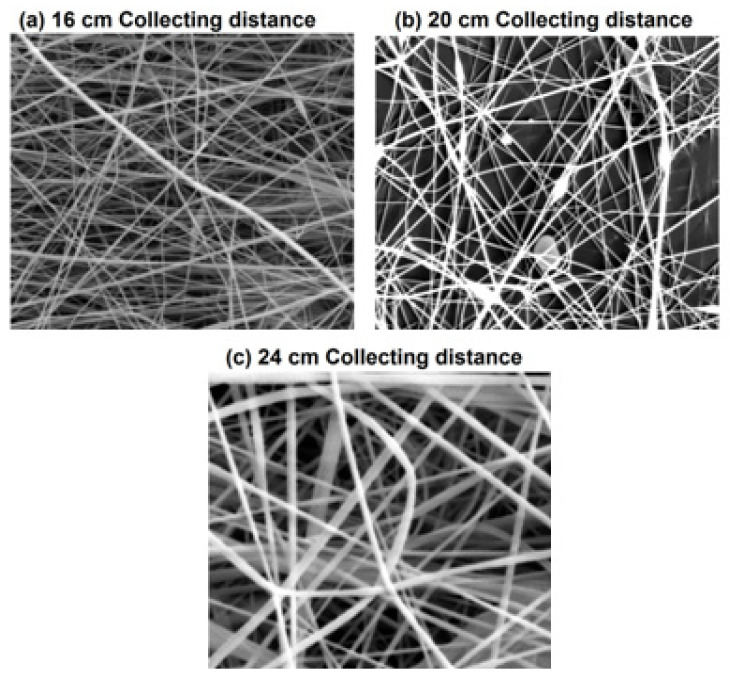
SEM images of nanofibers influencing the collecting distance 16 cm, 20 cm, and 24 cm at a constant voltage of 45 kV and with a constant polymer concentration of 20%.

**Figure 7:**
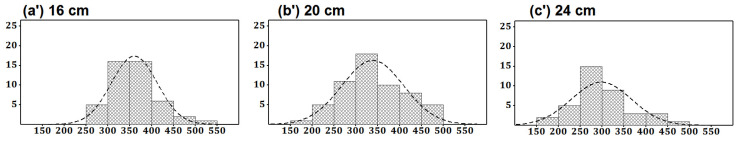
Histogram of nanofibers influencing the collecting distances 16 cm, 20 cm, and 24 cm at a constant voltage of 45 kV and with a constant polymer concentration of 20%.

Figure 6 and Figure 7 show SEM images and histograms of nanofibers influencing the collecting distance of 16, 20, and 24 cm at a constant voltage of 45 kV and polymer concentration of 20 *w*/*w*%. The SEM images of the PA nanofibers showed that increasing the collecting distance from 16 cm to 24 cm has an inverse effect on the diameter of nanofibers keeping the concentration and voltage at a constant level. Fibers with uniform distribution were obtained on shifting from 16 cm to 24 cm. It can also be observed that the impact of collecting distance is not very significant during the electrospinning process while keeping the distance at the spinning range.

**Figure 8:**
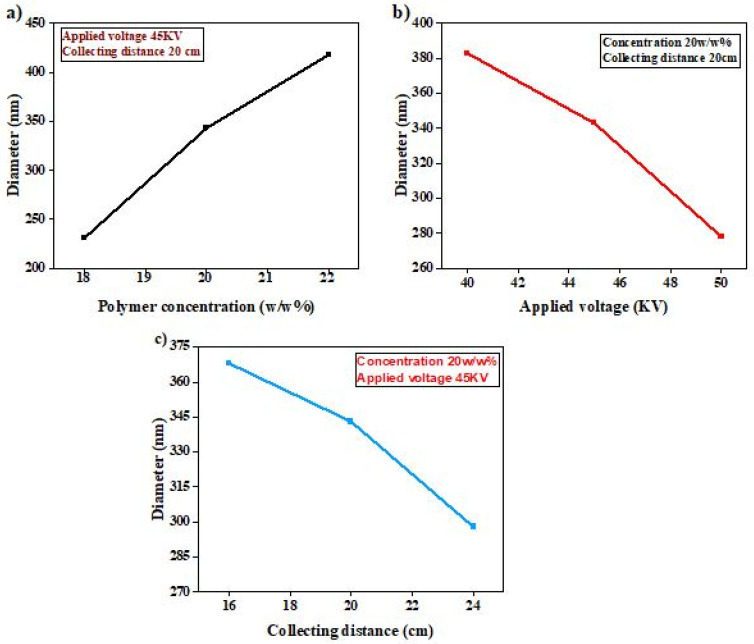
Relationship of nanofibers diameter, polymer concentration, and collecting distance.

The relation of nanofibers’ diameter with polymer concentration, voltage, and spinning distance is summarized in Figure 8. The increment in diameter of nanofibers with polymer concentration was found to be more significant as compared to the applied voltage and collecting distance. In contrast, by increasing the applied voltage from 40 kV to 50 kV, the diameter of nanofibers was decreased. Similarly, by increasing the collecting distance from 16 cm to 24 cm diameter of the nanofibers was decreased. As a result, bead-free and smoother fibers were obtained at 50 KV applied voltage, 20 *w*/*w*% concentration, and 24 cm collecting distance. Furthermore, these optimized nanomembranes were prepared at three different thickness levels, i.e., 80, 120, and 160 µm.

### 4.2. Surface Area and Porosity

**Figure 9:**
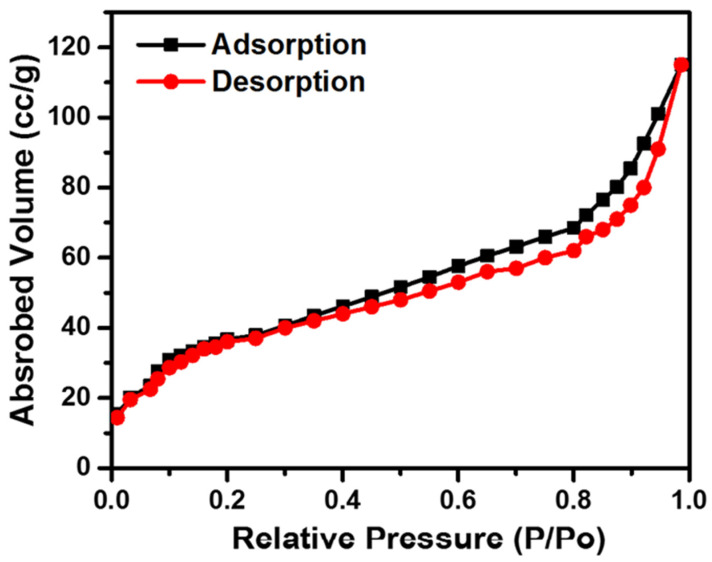
Nitrogen Adsorption–desorption isotherm curve of PA6 nanofiber membrane.

Figure 9 presents the nitrogen adsorption–desorption isotherm curve of the PA6 nanofiber membrane. The adsorption and desorption curves of the PA6 nanofiber follow the isotherm type IV behavior with the hysteresis of H3. PA6 nanofiber membrane exhibits a pore size of 12.416 nm, pore volume of along 0.081 cm^3^/g, and surface area of 36.043 m^2^/g. The pore size, pore volume, and surface area of the PA6 nanofiber membrane are very comparable, which can be very effective for biomedical applications.

### 4.3. Tensile and Bursting Strength of Nanomembranes

**Figure 10:**
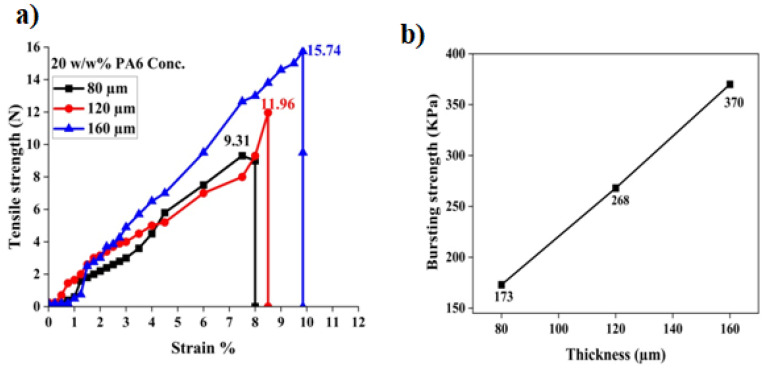
(**a**) Effect of nanofiber membrane thickness against the stress–strain curve. (**b**) Bursting strength of nanomembrane.

The tensile strength of nanomembranes of different thicknesses is given in Figure 10. The figure shows that tensile strength is increased by increasing the thickness of nanofibers. The nanofiber membranes with a thickness of 80, 120, and 160 µm revealed tensile strength of 9.31 ± 1.54, 11.96 ± 1.24, and 15.74 ± 0.88 N, respectively. This relation shows that by increasing thickness, the tensile strength of nanofibers also increases. When thickness changes from 80 µm to 120 µm, there is a 28.6% increase in the tensile strength of nanofibers that shows a significant improvement in strength. Similarly, when thickness changes from 120 µm to 160 µm, then there is a 31.6% increment in tensile strength. Moreover, the strain % of nanomembranes is also rising with an increase in thickness. The possible reason for this is the maximum no. of fibers and entanglement in the higher-thickness membrane as compared to the membrane with a low thickness.

Figure 10b shows the effect of the membrane’s thickness on the nanomembrane’s bursting strength. The membrane’s thicknesses of 80, 120, and 160 µm show bursting strengths of 173 ± 7.59, 268 ± 5.78, and 370 ± 4.15 KPa, respectively. It showed that when the thickness of the nanofibers membrane is increased, the bursting strength is also increased. This is because the interaction and entanglement of fibers increase with the increasing thickness of membranes. Each layer of membrane supports its neighboring membranes, due to which tensile and bursting properties of higher thickness membranes are better.

As hernia mesh needs higher tensile and bursting strength, the nanofiber membranes with a thickness of 160 µm were chosen to fabricate nanomembrane/textile composites.

### 4.4. Tear Strength Analysis of Composite and Standard Structures

**Figure 11:**
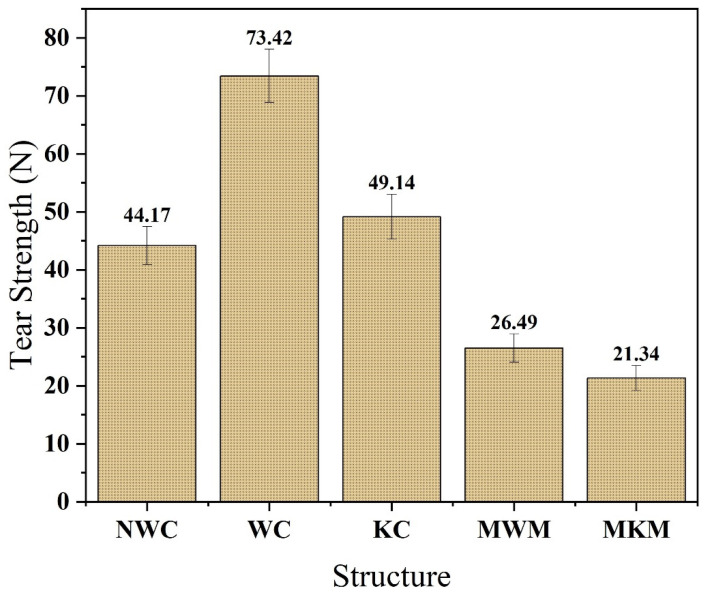
Tear strength of developed nanofiber membrane/textile composites and market-bought samples.

The calendaring process combined PA nanomembranes with non-woven, woven, and knitted fabrics. The comparison between the developed nanofiber/textile composite and the market-available mesh (knitted and woven) is shown in Figure 11. It was found that that market woven and knitted mesh was 26.49 ± 1.86 and 21.34 ± 1.98 N, respectively, but the tear strength of non-woven, woven, and knitted composites was 44.17 ± 1.86, 73.42 ± 1.54, and 49.14 ± 1.78 N, respectively. The woven composite showed much higher tear strength as compared to other structures. Knitted and non-woven composites showed relatively similar results. It was also observed that all the composite samples have greater tear strength than standard hernia mesh. The higher tear of the woven structure is due to its stable structure. The interlacement of yarn one on one makes the fabric very compact and stable. This leads to better tear strength as the interlacement hinders the propagation of the cut.

### 4.5. Bursting Strength Analysis of Composite and Standard Structures

**Figure 12:**
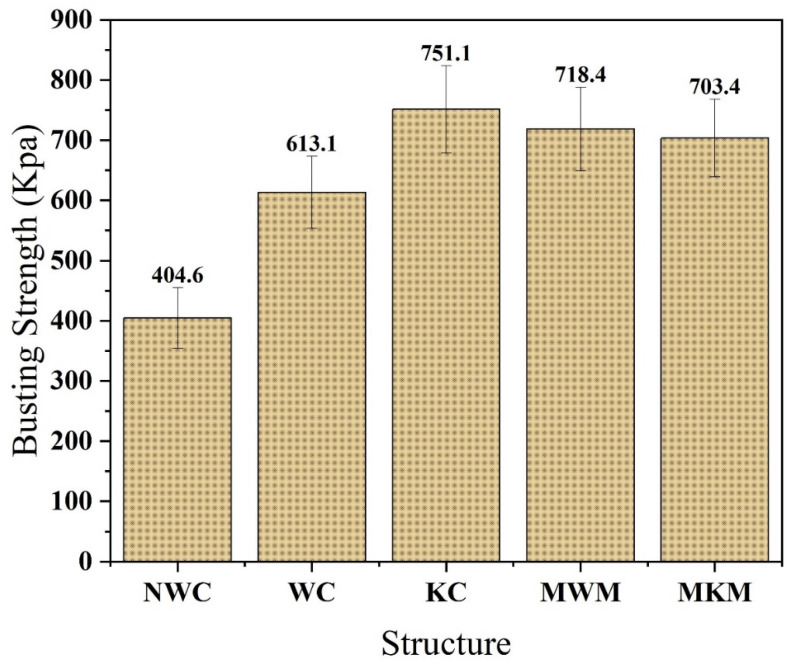
Bursting strength of developed nanofiber membrane/textile composites and market-bought samples.

Bursting is one of the most important mechanical properties that is used to measure how much a material can bear the force until it bursts. Figure 12 shows that the bursting strength of the standard woven and the knitted mesh was 718.4 and 703.4 KPa, respectively. However, the knitted, woven, and non-woven fabric-based composites were 751.1, 613.1, and 404.6 KPa, respectively. The knitted composite had the highest bursting strength compared to all other samples due to the knitted structure that gives elongation while stretching. In addition, when pneumatic pressure is applied, the knitted structure expands due to the loops structure and bears more force before bursting. Therefore, knitted-based composite can bear high blood pressure around the hernia inside the human body.

### 4.6. Tensile Strength Analysis of Composite and Standard Structures

**Figure 13:**
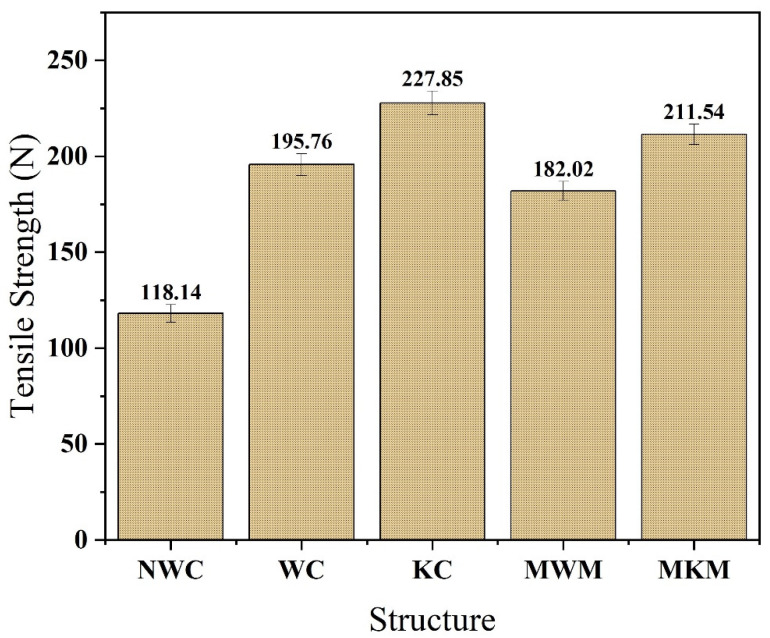
Tensile strength of developed nanofiber membrane/textile composites and market-bought samples.

Figure 13 shows the comparison of different electrospun nanofiber textile-supported composites by different fabric tensile strengths with reference meshes. The tensile strength of reference woven and knitted mesh is 182.02 and 211.54 N, respectively, which shows that reference knitted mesh had higher tensile strength than woven mesh. The tensile strength of non-woven, woven, and knitted-based composite is 118.14, 195.76, and 227.85 N, respectively, which shows that knitted fabric-supported nanofiber composite has the highest value of strength as compared to other developed composites. Knitted structures bear more force before breakage due to their loop structures. Loops absorb all initial force, and fabric elongates, breaking at a higher force. Therefore, knitted fabric-supported nanofiber composite was found most suitable in accordance with tensile and bursting strengths for repairing hernia disease due to being more resistive than other fabrics.

### 4.7. Elongation Properties Analysis of Composite and Standard Structures

**Figure 14:**
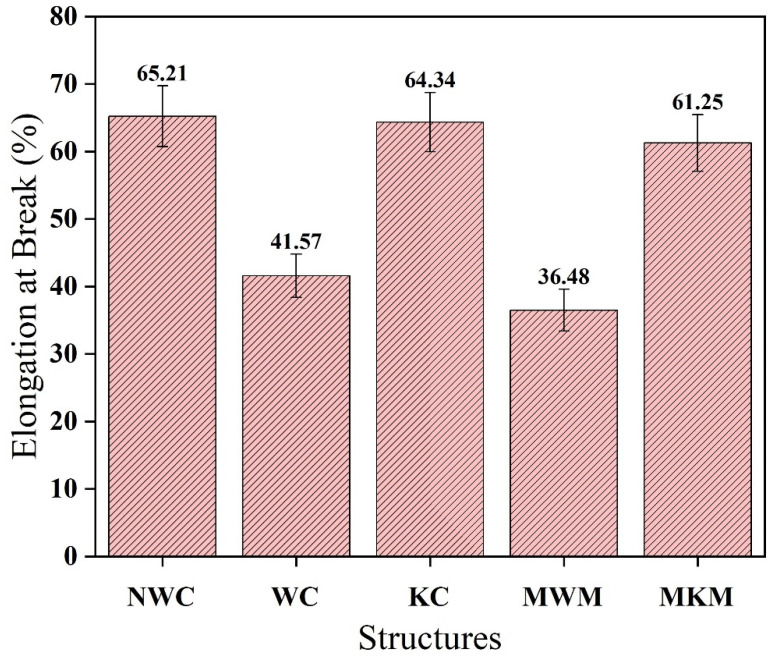
The comparison of elongation of developed nanofiber/textile composite with reference mesh.

The graph shown in Figure 14 explains the elongation properties of developed nanofiber/textile composite and reference hernia meshes. While looking at elongation %ages, reference woven mesh shows 36.48% elongation, and knitted mesh shows 58.22% elongation. Contrary to these meshes, self-developed nanofiber membrane/fabric-based composite shows different behavior. Self-developed woven composite elongation is 41.57%, while knitted-based composite is 68.34%, and the non-woven sample shows the least value of elongation, i.e., 29.78% after applying force. It is clear that self-developed nanofiber/textile composite showed better performance than market-available meshes. The knitted composite shows higher elongation due to the loop structure. When force is applied, loops of knitted structure elongate, and force is absorbed. Due to this reason, knitted-based composite has higher elongation. Interaction between fibers is least in non-woven structures, which results in less elongation. On applying force, fibers quickly slip into each other due to weak interaction and break earlier. Therefore, the tensile strength and elongation of non-woven are the least in all structures.

### 4.8. Stiffness of Composite and Standard Structures

**Figure 15:**
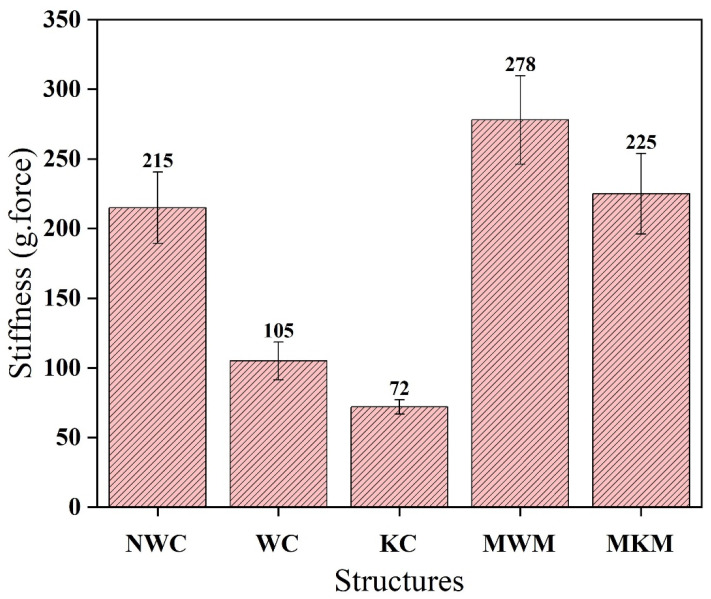
Comparison of the stiffness of composites developed against reference meshes.

The stiffness of composites developed against reference meshes is compared and shown in Figure 15. This shows that knitted, supported nanofiber composite has the lowest stiffness value compared to other developed composites. Non-woven composite gives the highest value of stiffness. The stiffness of woven fabric-supported composite was found between both structures. The stiffness of both reference meshes was much higher than the prepared nanofiber/textile composite. Knitted composite stiffness was the least due to its flexible behavior. This flexible structure is much more supportive for hernia support inside the human body. Therefore, the knitted composite is most suitable for this application.

### 4.9. Puncture Resistance of Composite and Standard Structures

**Figure 16:**
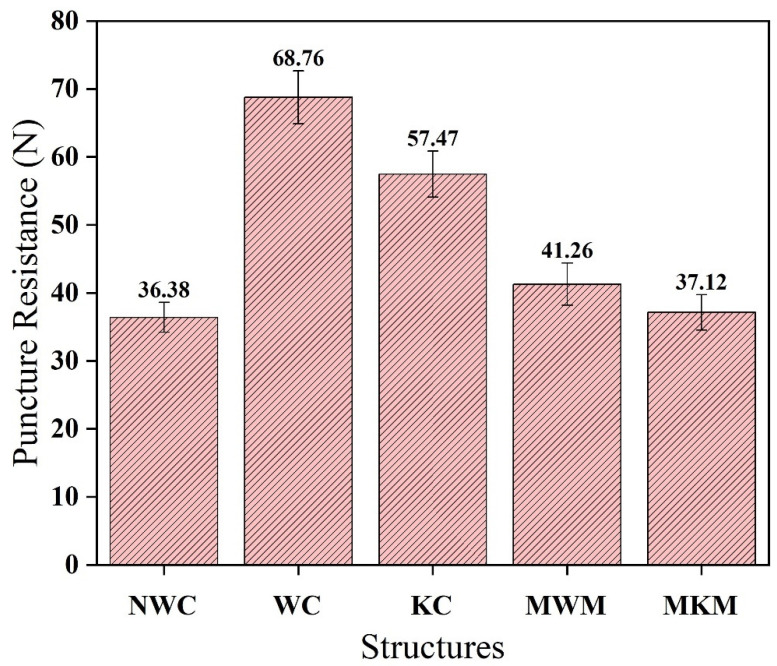
Comparison of puncture resistance of composites developed against reference meshes.

Figure 16 shows that the woven composite (WC) puncture resistance is higher than other fabric-based composite meshes. This behavior is due to its natural crisscross structure. All yarns are interlaced in a woven structure, which reduces the chances of puncture after applying pressure. Puncture resistance is also influenced by the material strength and cross-section of yarn. The greater the cross-section, the more puncture-resistant it is. The non-woven structure showed the least puncture resistance due to less interaction of constituent fibers. The puncture resistance of both reference samples is also less than composite structures. Knitted composite structures also showed impressive puncture resistance; hence, the knitted composite could be used as hernia mesh inside the human body.

## 5. Conclusions

In this research study, polyamide-6 nanofiber-based nanomembranes were synthesized using a needleless electrospinning process with the toothed wheel as a spinneret. Then different fabrics (woven, non-woven, knitted) were developed. Then the optimized nanofiber membrane was combined with each type of fabric to make a fabric-supported nanofiber composite. Based on characterizations, the morphology of the nanofiber membrane was affected by variations in applied voltage, polymer concentration, and collecting distance. Mechanical properties such as tensile, bursting, and tear properties of the PA nanofiber membrane were increased by increasing the thickness of nanomembranes. The tear strength and puncture resistance properties of the woven structure composite were much better as compared to other developed and reference meshes due to their stable structure. The knitted composite performed better in bursting strength, tensile, and stiffness properties than all other samples. It was concluded that hernia mesh should have high tensile strength, suture maintenance, excellent porosity to aid fibroblast colonization, minimum shrinkage tendency, stays flat without wrinkling, and have the correct design. When considering the obtained results, knitted composite has almost all these properties and was found to be potentially suitable for medical applications.

## Figures and Tables

**Table 1:** Optimization of electrospun nanofibers of PA.

Sr.	Concentration (*w*/*w*%)	Voltage (kV)	Distance (cm)
1.	18	40	16
2.	18	45	16
3.	18	50	16
4.	18	40	20
5.	18	45	20
6.	18	50	20
7.	18	40	24
8.	18	45	24
9.	18	50	24
10.	20	40	16
11.	20	45	16
12.	20	50	16
13.	20	40	20
14.	20	45	20
15.	20	50	20
16.	20	40	24
17.	20	45	24
18.	20	50	24
19.	22	40	16
20.	22	45	16
21.	22	50	16
22.	22	40	20
23.	22	45	20
24.	22	50	20
25.	22	40	24
26.	22	45	24
27.	22	50	24

## Data Availability

Data will be available upon request.

## References

[1] Rigby A.J., Anand S.C., Horrocks A.R. (1997). Textile Materials for Medical and Healthcare Applications. J. Text. Inst..

[2] Brocket J., Shaban R.Z. (2015). Characteristics of a successful hospital hand hygiene program: An Australian perspective. Healthc. Infect..

[3] Burnett-Boothroyd S., McCarthy B. (2011). Antimicrobial treatments of textiles for hygiene and infection control applications: An industrial perspective. Textiles for Hygiene and Infection Control.

[4] Ahmad A., Ali U., Nazir A., Shahzad A., Khaliq Z., Qadir M.B., Khan M.A., Ali S., Hassan M.A., Abid S. (2019). Toothed wheel needleless electrospinning: A versatile way to fabricate uniform and finer nanomembrane. J. Mater. Sci..

[5] Shekar K., Mullany D.V., Thomson B., Ziegenfuss M., Platts D.G., Fraser J.F. (2014). Extracorporeal life support devices and strategies for management of acute cardiorespiratory failure in adult patients: A comprehensive review. Crit. Care.

[6] Joung Y.-H. (2013). Development of Implantable Medical Devices: From an Engineering Perspective. Int. Neurourol. J..

[7] Iliadis D.P., Bourlos D.N., Mastrokalos D.S., Chronopoulos E., Babis G.C. (2016). LARS Artificial Ligament versus ABC Purely Polyester Ligament for Anterior Cruciate Ligament Reconstruction. Orthop. J. Sports Med..

[8] Giacone D.V., Dartora V.F., de Matos J.K., Passos J.S., Miranda D.A., de Oliveira E.A., Silveira E.R., Costa-Lotufo L.V., Maria-Engler S.S., Lopes L.B. (2020). Effect of nanoemulsion modification with chitosan and sodium alginate on the topical delivery and efficacy of the cytotoxic agent piplartine in 2D and 3D skin cancer models. Int. J. Biol. Macromol..

[9] Ludwicka A., Jansen B., Wadström T., Pulverer G. (1984). Attachment of staphylococci to various synthetic polymers. Zent. Für Bakteriol. Mikrobiol. und Hygiene. 1. Abt. Originale. A Med. Mikrobiol. Infekt. und Parasitol..

[10] Ledet E.H., Liddle B., Kradinova K., Harper S. (2018). Smart implants in orthopedic surgery, improving patient outcomes: A review. Innov. Entrep. Health.

[11] Hinton R.B., Yutzey K.E. (2011). Heart valve structure and function in development and disease. Annu. Rev. Physiol..

[12] Lockhart K., Dunn D., Teo S., Ng J.Y., Dhillon M., Teo E., van Driel M.L. (2018). Mesh versus non-mesh for inguinal and femoral hernia repair. Cochrane Database Syst. Rev..

[13] Baylón K., Rodríguez-Camarillo P., Elías-Zúñiga A., Díaz-Elizondo J.A., Gilkerson R., Lozano K. (2017). Past, Present and Future of Surgical Meshes: A Review. Membranes.

[14] Melman L., Jenkins E.D., Hamilton N.A., Bender L.C., Brodt M.D., Deeken C.R., Greco S.C., Frisella M.M., Matthews B.D. (2011). Histologic and biomechanical evaluation of a novel macroporous polytetrafluoroethylene knit mesh compared to lightweight and heavyweight polypropylene mesh in a porcine model of ventral incisional hernia repair. Hernia.

[15] Usher F.C. (1982). Method of Hernia Repair. U.S. Patents.

[16] Roeber P. J. (2009). Knit PTFE Articles and Mesh. U.S. Patent.

[17] Kalliomäki M.-L., Meyerson J., Gunnarsson U., Gordh T., Sandblom G. (2008). Long-term pain after inguinal hernia repair in a population-based cohort; risk factors and interference with daily activities. Eur. J. Pain.

[18] Stoppa R. (1998). Hernia of the abdominal wall. Hernias and Surgery of the Abdominal Wall.

[19] Ali M., Zeeshan M., Ahmed S., Qadir M.B., Nawab Y., Anjum A.S., Riaz R. (2018). Development and Comfort Characterization of 2D-Woven Auxetic Fabric for Wearable and Medical Textile Applications. Cloth. Text. Res. J..

[20] Mitura K., Romańczuk M. (2008). Valenti method (PAD) as an assesment of polypropylene mesh fixing standarization in inguinal hernia repair. Folia Med. Crac..

[21] Ramshaw B., Forman B.R., Heidel E., Barker E. (2019). Laparoscopic Ventral Hernia Repair with a Non-Woven Hernia Mesh. Surg. Technol. Online.

[22] Khanzada H., Salam A., Qadir M.B., Phan D.-N., Hassan T., Munir M.U., Pasha K., Hassan N., Khan M.Q., Kim I.S. (2020). Fabrication of Promising Antimicrobial Aloe Vera/PVA Electrospun Nanofibers for Protective Clothing. Materials.

[23] Wu S., Dong T., Li Y., Sun M., Qi Y., Liu J., Kuss M.A., Chen S., Duan B. (2022). State-of-the-art review of advanced electrospun nanofiber yarn-based textiles for biomedical applications. Appl. Mater. Today.

[24] Wei L., Sun R., Liu C., Xiong J., Qin X. (2019). Mass production of nanofibers from needleless electrospinning by a novel annular spinneret. Mater. Des..

[25] Ebersole G.C., Buettmann E.G., MacEwan M.R., Tang M.E., Frisella M.M., Matthews B.D., Deeken C.R. (2012). Development of novel electrospun absorbable polycaprolactone (PCL) scaffolds for hernia repair applications. Surg. Endosc..

[26] Molnár K., Voniatis C., Fehér D., Ferencz A., Fónyad L., Reiniger L., Zrinyi M., Wéber G., Jedlovszky-Hajdu A. (2018). Biocompatibility study of poly (vinyl alcohol)-based electrospun scaffold for hernia repair. Express Polym. Lett..

[27] Liu P., Chen N., Jiang J., Wen X. (2019). New surgical meshes with patterned nanofiber mats. RSC Adv..

[28] Veleirinho B., Coelho D.S., Dias P.F., Maraschin M., Pinto M.R.R., Cargnin-Ferreira E., Peixoto A., Souza J.A., Ribeiro-Do-Valle R.M., Lopes-Da-Silva J.A. (2014). Foreign Body Reaction Associated with PET and PET/Chitosan Electrospun Nanofibrous Abdominal Meshes. PLoS ONE.

[29] Chakroff J., Kayuha D., Henderson M., Johnson J. (2015). Development and Characterization of Novel Electrospun Meshes for Hernia Repair. SOJ Mater. Sci. Eng..

[30] Plencner M., Prosecká E., Rampichová M., East B., Buzgo M., Vysloužilová L., Hoch J., Amler E. (2015). Significant improvement of biocompatibility of polypropylene mesh for incisional hernia repair by using poly-ε-caprolactone nanofibers functionalized with thrombocyte-rich solution. Int. J. Nanomed..

[31] Ali U., Abbass A., Khurshid F., Aslam S., Waqar A. (2017). Needleless Electrospinning Using a Flat Wheel Spinneret. J. Eng. Fibers Fabr..

[32] Zhao Y., Wang X., Wang D., Li H., Li L., Zhang S., Zhou C., Zheng X., Men Q., Zhong J. (2020). Preparation and Chemical Protective Clothing Application of PVDF Based Sodium Sulfonate Membrane. Membranes.

[33] Sun K.C., Arbab A.A., Sahito I.A., Qadir M.B., Choi B.J., Kwon S.C., Yeo S.Y., Yi S.C., Jeong S.H. (2017). A PVdF-based electrolyte membrane for a carbon counter electrode in dye-sensitized solar cells. RSC Adv..

[34] Gikunda M.N., Harerimana F., Mangum J.M., Rahman S., Thompson J.P., Harris C.T., Churchill H.O.H., Thibado P.M. (2022). Array of Graphene Variable Capacitors on 100 mm Silicon Wafers for Vibration-Based Applications. Membranes.

[35] Tang Y., Zhong L., Wang W., He Y., Han T., Xu L., Mo X., Liu Z., Ma Y., Bao Y. (2022). Recent Advances in Wearable Potentiometric pH Sensors. Membranes.

[36] Goh L.M., Thong Z., Li W.P., Ooi S.T., Esa F., Ng K.S., Dhalla A., Gudipati C. (2022). Development and Industrial-Scale Fabrication of Next-Generation Low-Energy Membranes for Desalination. Membranes.

[37] Wang X., Lin T., Wang X. (2014). Scaling up the production rate of nanofibers by needleless electrospinning from multiple ring. Fibers Polym..

